# Coronavirus nucleocapsid proteins: a multifaceted modulator in the innate immune evasion

**DOI:** 10.3389/fmicb.2025.1658339

**Published:** 2025-12-09

**Authors:** Yuting Xiao, Ziyao Song, Lei Zhou, Wanqing Lu, Weihuan Fang, Jidong Xu, Xiaoliang Li

**Affiliations:** 1 Department of Veterinary Medicine, College of Animal Sciences, Institute of Preventive Veterinary Medicine, Zhejiang University, Hangzhou, Zhejiang, China; 2 ZJU-Xinchang Joint Innovation Centre (TianMu Laboratory), Shaoxing, Zhejiang, China; 3 The Rural Development Academy, Zhejiang University, Hangzhou, Zhejiang, China

**Keywords:** coronaviruses, nucleocapsid protein, innate immunity, immune evasion, pathogenesis

## Abstract

Coronaviruses are capable of inducing diverse infectious diseases that pose significant threats to the public health and the economic development. With a single positive-stranded RNA genome, coronaviruses utilize viral proteins to execute diverse immune escape strategies to facilitate their replication. Of all the identified structural proteins and non-structural proteins within the coronaviruses, nucleocapsid (N) protein is highly conserved and is the most abundant viral protein in infected host cells. N protein regulates the more complex and diverse mechanisms through which viruses suppress host immunity. In this review, we analyzed the basic structure of coronavirus N protein, and further elaborate on its multifaceted regulatory functions in the virion assembly, pathogenesis, host innate immune responses, as well as the innate immunity-related programmed cell death and cell cycle, and also other cell processes. A better understanding of the immune evasion strategy regulated by N protein will help to provide a theoretical basis for the development of broad-spectrum anti-coronavirus drugs targeting N proteins.

## 1 Introduction

Coronavirus is a class of enveloped single-stranded positive-sense RNA viruses belonging to the family *Coronaviridae* of the order *Nidovirales*. Also, the coronaviruses represent as the largest known RNA viruses identified to date (Lin et al., 2020). Based on the distinct serological properties and genomic features, this virus family is systematically classified into four major genera: Genus *Alphacoronavirus*, including human coronavirus 229E (HCoV-229E), porcine epidemic diarrhea virus (PEDV), feline infectious peritonitis virus (FIPV), and other viruses that infect mammals; Genus *Betacoronavirus*, including severe acute respiratory syndrome coronavirus (SARS-CoV), middle east respiratory syndrome coronavirus (MERS-CoV), severe acute respiratory syndrome coronavirus 2 (SARS-CoV-2), and mouse hepatitis virus (MHV); Genus *Gammacoronavirus*, such as avian infectious bronchitis virus (IBV); and Genus *Deltacoronavirus*, such as porcine deltacoronavirus (PDCoV) (Woo et al., 2012; Anderson et al., 2024; Liu et al., 2023; Xue et al., 2024). With the characteristics of high pathogenicity and contagiosity, these coronaviruses cause severe respiratory and intestinal epidemic diseases in birds and mammals, posing significant threats to the global public health and veterinary medicine (Zhou et al., 2020).

**Figure 1:**
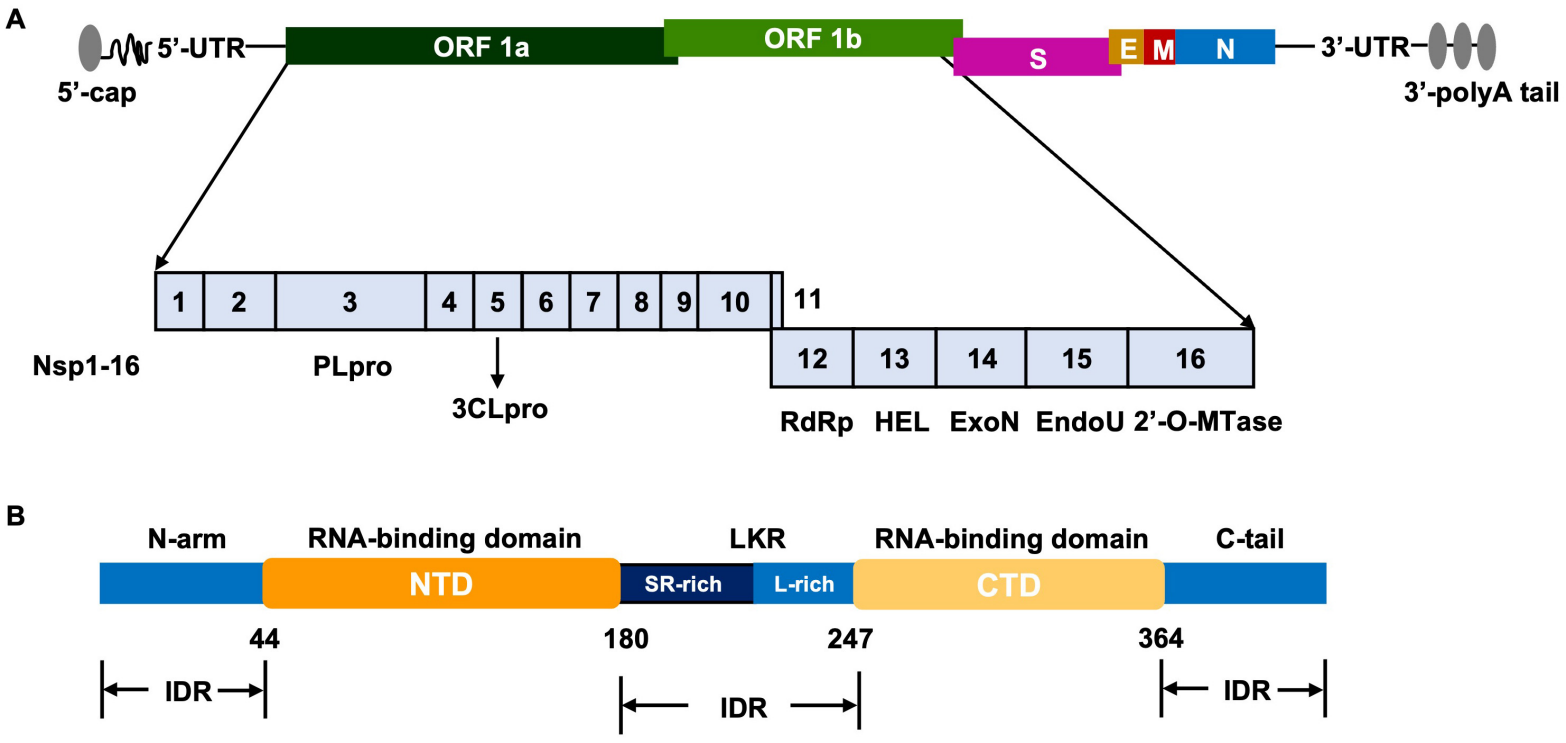
Coronavirus RNA genomic structure **(A)** and Cornoavirus N protein structure **(B)**. NTD, N-terminal domain; CTD, C-terminal domain; IDR, intrinsically disordered region.

The coronavirus genome is approximately 26–32 kb in length, featuring a 5′ cap structure and a 3′ poly(A) tail. Its organization includes a 5′ untranslated region (UTR), several open reading frames (ORFs), and a 3′ UTR (Malik, 2020). The 5′-terminal ORF1a and ORF1b encode the pp1a and pp1ab polyproteins, which are cleaved into 16 non-structural proteins (Nsp1-16) that form the viral replication-transcription complex. The downstream ORFs encode the four essential structural proteins-Spike (S), Envelope (E), Membrane (M), and Nucleocapsid (N)-as well as a set of accessory proteins (e.g., those from ORF3) which are not cleaved into Nsps and often play roles in virus-host interaction shown in Figure 1A (Li X. et al., 2020). The structural and nsps encoded by each gene play crucial roles in the life cycle of coronaviruses (Beall et al., 2016). The S protein contains several N-glycosylation sites and is associated with viral pathogenicity and immunogenicity by binding to specific cell membrane receptors and mediate viral attachment, membrane fusion and invading host cells (Kirchdoerfer et al., 2016; Song et al., 2004). The E protein is involved in the assembly of the viral particles, similar to the function of M protein (Fehr and Perlman, 2015). The N protein is a highly conserved viral protein and is dynamically associated with viral RNA replication sites by encapsulating the viral RNA genome; it also plays a role in protecting the viral genome and mediating its transfer to the budding site. The nsps interact with each other to form the RNA replicase-transcriptase complex, which regulates viral RNA replication and coordinates the transcription of subgenomic RNAs (Liu et al., 2021; Zeng et al., 2020). Additionally, the nsps play crucial roles in the whole life cycle of coronaviruses, by regulating viral RNA replication and transcription, viral assembly and release, and also different host cell processes for the viral immune evasion (Snijder et al., 2016).

It is worth mentioning that both the viral structural proteins and the nsps can interact and regulate a series of host proteins for evading the host antiviral immune response and exploiting the host cell to facilitate viral proliferation. As N protein is the most abundantly expressed protein in coronaviruses and also one of the most extensively studied proteins in the context of innate immune evasion (Bai et al., 2021), we focus on describing the multifaceted functions on it for a better understanding of coronavirus pathogenesis.

## 2 Basic structure of the coronavirus N protein

Coronavirus N protein is a highly conserved structural protein and plays a vital role in the viral life cycle (Lin et al., 2022). The molecular weight of N protein typically ranges between 43–50 kDa, with specific sizes varying depending on different coronavirus species (Kadam et al., 2021). The N protein usually exists as a dimer or higher-order oligomer in viral particles, which is critical for its multiple functions (McBride et al., 2014). Moreover, based on the sequence alignment and functions of the N proteins from different coronaviruses, it is generally composed of two main structural domains: the N-terminal domain (NTD) and the C-terminal domain (CTD), connected by a linker region of variable length shown in Figure 1B (Chang et al., 2014). The NTD often contains a conserved folding structure with RNA-binding activity and mainly functions in binding viral RNA, forming the ribonucleoprotein (RNP) complex (Huang et al., 2004; Lan et al., 2020). The CTD is a self-binding domain containing a dimerization interface that promotes N protein dimerization and regulates N protein interacting with other viral structural proteins (Masters, 2019). The linker region connects the NTD and CTD within the N protein, providing flexibility that allows relative movement between the two domains (Kang et al., 2020). Recently, Morse et al. developed an optical tweezers-based assay to directly observe, in real time, how SARS-CoV-2 N protein binds and compacts RNA into virus-like structures at single-molecule resolution and revealed that NTD initiates RNA binding while CTD mediates oligomerization, together driving the formation of ultra-compact ribonucleoprotein complexes essential for viral packaging by constructing truncated variants (Morse et al., 2023). By utilizing the electron microscopy, biochemical/biophysical techniques together with molecular modeling and molecular dynamics simulations, Ribeiro-Filho et al. (2022) demonstrated that SARS-CoV-2 N protein transitions from dynamic extended dimers to compact RNA-bound octamers, revealing a conformational plasticity essential for its RNA packaging function, potentially indicating the conformational plasticity and functional versatility of coronavirus N protein.

In addition, coronavirus N protein contains several intrinsically disordered regions (IDRs), which can undergo conformational changes upon ligand binding and is crucial for binding RNA or other proteins (Figure 1B) (Nguyen et al., 2024). It has been reported that IDRs within the NTD of coronavirus N protein can be phosphorylated and can regulate the liquid-liquid phase separation dynamics by modulating RNA-responsive behavior and maintaining droplet fluidity, providing mechanistic insights into how N-terminal mutations may affect viral assembly (Zachrdla et al., 2022; Wang et al., 2021). Also, IDRs within the N protein is a key role in regulating the extensive genetic diversity of coronaviruses, particularly by modulating protein stability, and oligomerization (Nguyen et al., 2024). More importantly, the N protein is an alkaline phosphoprotein with multiple potential posttranscriptional modification sites, by which N protein regulates its interactions with genome RNA or other viral/host proteins, also affects the N protein stability and functions in a wide range of complex biological processes (Fung and Liu, 2018).

Therefore, these structural features determine the multifunctional characteristic of N protein both in the viral pathogenesis and host antiviral innate immunity. It is crucial for developing antiviral drugs and vaccines to understand these structure-function relationships.

## 3 Function diversity of coronavirus N protein by disrupting host immune responses

### 3.1 N protein in virion assembly and pathogenesis

As is mentioned above, the coronavirus N protein serves as a multifunctional protein with a complex structure and multiple functional domains, thus plays a critical role in the viral assembly and pathogenesis.

The N protein binds viral RNA via the RNA-binding domain (RBD) in its N-terminal domain (NTD) to form ribonucleoproteins (RNPs), a critical step for genome packaging and the accurate incorporation of the viral genome into new particles (Kuo et al., 2014; Korn et al., 2023). Beyond this structural role, the efficiency of subsequent assembly and budding constitutes a key innate immune evasion strategy. By ensuring rapid virion formation and egress, the N protein minimizes the cytosolic exposure of viral nucleic acids (Li et al., 2020). This process reduces their detection by intracellular pattern recognition receptors such as RIG-I and MDA5, thereby suppressing the initiation of the type I interferon response (Li et al., 2020; Kopecky-Bromberg et al., 2007). Also, the N protein is capable of forming dimers by oligomerization which enhances its RNA-binding capacity and facilitates the RNP complexes formation, thereby prompt the structural integrity of viral particles (Zhao et al., 2024).

Furthermore, as coronavirus M protein is essential for viral assembly (Zhang et al., 2022), N protein interacts with the M protein by the CTD to recruit the RNP complexes to the correct viral assembly sites and promote virion assembly and budding (Kuo et al., 2016; Siu et al., 2008; Boscarino et al., 2008). In the year of 2004, He et al. (2004a,b) investigated potential interaction between SARS-CoV N protein and the M protein by mammalian two-hybrid system, and found that the amino acids (168–208) within the N protein should be the key binding motif for the N protein-M protein interaction, which is necessary for N protein multimerization and correct conformation of N protein. Furthermore, coronavirus N protein interacts with the Ubl1 domain of its replicase-transcriptase subunit Nsp3 by its SR region shown in Figure 1B, which is critical to the RNA synthesis and initiation (Koetzner et al., 2022).

Coronavirus N protein also plays a regulatory role in the viral pathogenesis to evade host innate immune systems for viral replication by modulating the host innate immune responses, virus-host cells interaction and inflammatory responses. These functions make N protein a vital molecule in the coronavirus life cycle and pathogenic mechanisms.

### 3.2 Coronavirus N protein functions as the IFN antagonist by disrupting the related signaling pathways

The innate immune response is the host initial barrier in against pathogen invasion. Innate immune cells recognize pathogen-associated molecular patterns (PAMPs) through pattern recognition receptors (PRRs), thereby activating anti-infection signaling pathways and stimulating the production of cytokines and chemokines (Kasuga et al., 2021). PRRs that play a key role in the process of coronavirus infection mainly include retinoic acid-inducible gene I-like receptors (RLRs) and Toll-like receptors (TLRs), which effectively inhibit viral replication and transmission by recognizing viral RNA and activating downstream signaling. The feedback mechanism of interferon (IFN) as a key antiviral cytokine involves multilevel signaling (Yoneyama et al., 2015; Kawai and Akira, 2008). Followed that RLR family members RIG-I and MDA5 recognize the viral RNA, they form complexes with mitochondrial antiviral signaling protein (MAVS) and tumor necrosis factor receptor-associated factor 3 (TRAF3), which in turn activate IKKε/TBK1 kinase, promote phosphorylation of IFN regulators IRF3 and IRF7, and induce the production of type I IFNs. On the other hand, TLRs synergistically promote the expression of type I IFNs and inflammatory cytokines by activating NF-κB, MAPK signaling pathways, and IRF3 through MyD88 and TRIF adaptor proteins after recognizing viral RNA (Li S. et al., 2020). By binding to the target cell surface receptors, the secreted IFN activates the JAK-STAT signaling cascade and induces the expression of hundreds of interferon-stimulated genes (ISGs), forming a strong antiviral defense network, significantly inhibiting viral replication and blocking its diffusion process.

However, coronaviruses can antagonize the whole antiviral IFN signaling pathway in multiple ways by using their N proteins. In the early stage of IFN signaling activation, Hu et al. found that SARS-CoV and MERS-CoV N proteins interact with TRIM25 and inhibit RIG-I signaling, which in turn inhibit type I IFN production (Hu et al., 2017). Chang et al. also demonstrated that MERS-CoV N protein can act as an IFN antagonist, and its interaction with TRIM25 blocks the ubiquitination of RIG-I and the activation of the RIG-I pathway. During its infection cycle and transmission, N protein of SARS-CoV-2, a highly pathogenic human coronavirus, induces a complex network of host antiviral responses. Zheng et al. showed that the interaction of SARS-CoV-2 N protein with G3BP1 attenuates the formation of antiviral stress particles (avSG) and prevents cofactors G3BP1 and PACT from activating RIG-I, thereby inhibiting RIG-I/MDA5-mediated type I and III IFN responses. Furthermore, N protein also affects the RIG-I-mediated dsRNA recognition (Zheng et al., 2022). SARS-CoV N proteins exert IFN antagonism by targeting protein activators (PACT) of interferon-induced protein kinases and impairing PACT-RIG-I/MDA5 interactions (Ding et al., 2017). IBV N interferes with MDA5 recognition of double-stranded RNA (dsRNA) and targets LGP2, hindering the activation of MDA5 and LGP2-stimulated IFN-β promoters, inhibiting type I IFN production, and thereby inhibiting the host's innate immune response (Huang et al., 2023). Since RIG-I signaling triggers phosphorylation and translocation of key transcription factors, including NF-κB and IRF3, phosphorylation of IRF3 and NF-κB p65 subunits is attenuated in the presence of MERS-CoV N protein, resulting in inhibition of IFN production (Chang et al., 2020). Guo et al. found that SARS-CoV-2 N protein inhibits the activation of the NF-κB pathway and its downstream signaling by blocking the assembly of the TAK1-TAB2/3 complex through its CTD region, and therefore, SARS-CoV-2 N inhibits the occurrence of cellular inflammation and promote viral replication. In addition, Glu-290 and Gln-349 within the SARS-CoV-2 N protein CTD are key sites that inhibit the NF-κB pathway (Guo et al., 2025). SARS-CoV N protein interacts with the host transcription factor NF-κB to regulate interleukin-6 (IL-6) expression and induce innate inflammatory response (Zhang et al., 2007). TGEV N protein enhances neonatal Fc receptor (FcRn) promoter activity by activating the NF-κB signaling pathway to resist viral invasion in intestinal mucosal immunity; in addition, N-protein-mediated upregulation of FcRn promotes IgG transcytosis (Qian et al., 2019). However, to date, there is no evidence indicating that TGEV N protein affects IFN-β production. Although it has been reported that PEDV N protein can activate NF-κB through the TLR2, TLR3, and TLR9 pathways in porcine intestinal epithelial cells (Cao et al., 2015), Ding Z. et al. (2014) found PEDV N protein competitively inhibited the interaction between IRF-3 and TBK1 by targeting TANK-binding kinase 1 (TBK1), blocking the production of IFN-β by host cells. It also antagonizes IFN-λ production by preventing the transcription factor NF-κB nuclear translocation. Coronavirus N proteins can also block the downstream of IFN signaling by suppressing JAK-STAT signaling and the related ISGs expression. For example, as the only member of deltacoronavirus, PDCoV prevents the activation of the JAK-STAT signaling pathway by interacting with STAT1 through N protein interaction with STAT1 and inhibiting nuclear translocation of STAT1 by degrading KPNA2 (Hu et al., 2024). As histone deacetylase 1 (HDAC1) positively regulates the transcription of two antiviral IFN-stimulated genes (ISGs), ISG15 and OAS1, PEDV N protein impairs their expression by competitively binding to the transcription factor Sp1 and blocking the transcription of HDAC1 in the nucleus to disrupt STAT1 acetylation-phosphorylation balance, resulting in the inhibition of JAK-STAT1 signaling and facilitation of viral replication (Xu et al., 2021).

**Figure 2:**
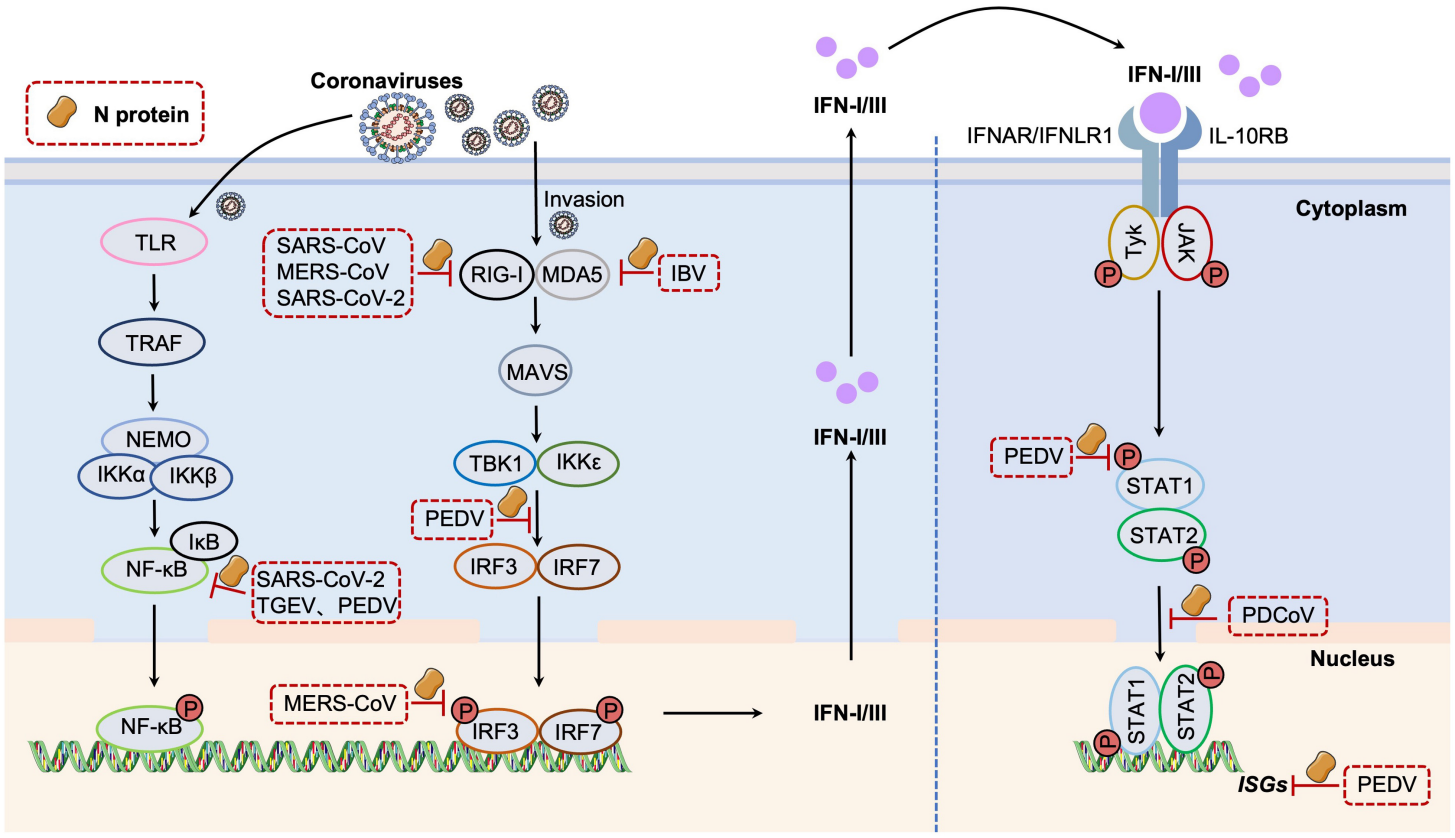
Coronavirus N protein in inhibiting host IFN signaling pathway.

In summary, coronavirus N proteins employ a multifaceted strategy to evade the innate immune response by targeting two critical junctures: the initial detection of viral RNA and the subsequent signaling cascades. They disrupt upstream pathogen recognition by interfering with RLRs (RIG-I and MDA5) and their co-factors (TRIM25, PACT, G3BP1), thereby preventing the activation of key transcription factors like IRF3 and NF-κB. Simultaneously, they inhibit downstream signaling pathways, including the JAK-STAT pathway, the TBK1-IRF3 axis, and the NF-κB pathway, to block the production of interferons, inflammatory cytokines, and antiviral ISGs. This coordinated suppression of both the induction and effector phases of the interferon response is a central mechanism of coronavirus immune evasion (Figure 2). It is worth mentioning that, as the final key effectors in the IFN signaling transduction process, different ISGs target the coronavirus N proteins for their efficient antiviral functions (e.g., ISGylation) (Bang et al., 2024; Zhu et al., 2024).

However, there is few studies showing how N proteins exerts immune evasion by interacting these ISGs. Further study may be driven by this direction for a more comprehensive understanding of the pathogenic mechanism of coronaviruses. (Q3,Q5)

### 3.3 N protein in antiviral immunity-related programmed cell death

#### 3.3.1 Autophagy

Autophagy is an evolutionarily conserved biological process of intracellular degradation that can be activated in response to a variety of cellular stress signals, such as nutrient deficiencies, hypoxia, endoplasmic reticulum stress, PAMPs stimulation, and pathogen invasion (Kong et al., 2020). This process exerts an antiviral effect through the selective degradation of viral proteins: firstly, E3 ubiquitin ligase recognizes and modifies the viral proteins for ubiquitination, followed by cargo-specific autophagy receptors recognition and directing to the autophagolysosomal system for degradation, thereby effectively inhibiting viral replication (Deretic et al., 2013; Levine et al., 2011; Ko et al., 2017). Several studies have confirmed that autophagy, as a core component of the host defense system, can directly degrade viral particles through lysosome-dependent pathways and interfere with the viral life cycle (Kim et al., 2010). However, the game between viruses and hosts is complex-although the host inhibits viral replication through autophagy degradation of targeted viral proteins, some viruses have evolved reverse manipulation strategies to hijack the autophagic pathway to degrade host antiviral proteins, thereby weaken the innate immune responses and promoting self-proliferation.

On one hand, autophagy serves as a potent host defense by directly targeting viral proteins for degradation. This antiviral arm of selective autophagy is often initiated by host antiviral factors. For instance, cytoplasmic PABPC4 can act as a bridge, recruiting the N proteins of multiple coronaviruses (including SARS-CoV-2, SARS-CoV, MERS-CoV, PDCoV, and IBV) to the autophagy receptor NDP52 for subsequent lysosomal degradation, thereby suppressing viral replication (Jiao et al., 2021). HNRNPA1 is a novel host limiting factor that interacts with many viral proteins and regulates viral replication (Ríos-Marco et al., 2016). HNRNPA1 activates the HNRNPA1-MARCHF8/MARCH8-CALCOCO2/NDP52-autophagosome pathway with the assistance of the transcription factor EGR1and IFN-induced antiviral immunity to protect the host from virus damage. The above studies show that selective autophagy plays a dual role in PEDV infection and host innate immunity. Kong et al. demonstrated that bone marrow stromal cell antigen 2 (BST2) inhibits PEDV replication by selectively autophagy binding and degradation of the PEDV-encoded N protein (Kong et al., 2020). Yang et al. (2023) demonstrated that the phosphoglycerate mutase PGAM5 degrades PDCoV through autophagy and activates IFN-I to antagonize viral replication by interacting with the transport receptors P62 and E3 ubiquitination ligases STUB1. Ji et al. (2024) revealed a novel mechanism by which the E3 ubiquitin ligase FBXW8 inhibits viral proliferation through NPD52-dependent autophagic degradation of the PDCoV N protein. In the chicken macrophage HD_11_ cell line, chicken guanylate-binding protein 1 (chGBP1) interacts with IBV N and inhibits viral replication by degrading N protein through the autophagic pathway (Ma et al., 2023). In these scenarios, autophagy benefits the host when it is successfully mobilized to recognize and destroy viral components, effectively curbing the infection.

On the other hand, coronaviruses, particularly through their N protein, can strategically manipulate the autophagic pathway to evade immunity and promote replication. For example, the SARS-CoV-2 N protein can induce autophagy by inhibiting mTORC1 through its interaction with LARP1 (Gordon et al., 2020). More directly, the PEDV N protein hijacks the autophagy machinery to degrade the antiviral host protein HNRNPA1, thereby dismantling a critical defense barrier (Zhai et al., 2023). Similarly, the interaction between PEDV N and TRIM28 induces mitophagy, which leads to the inhibition of the JAK-STAT1 signaling pathway and enhanced viral replication (Li et al., 2024). The localization of TGEV N protein to mitochondria, inducing mitophagy and damage, also points to a viral strategy to disrupt cellular homeostasis (Zhu et al., 2016). Therefore, autophagy benefits the virus when viral proteins like N succeed in repurposing it to degrade host antiviral factors, disrupt immune signaling, and generate replication-friendly environments.

Thus, autophagy is a “double-edged sword” in viral replication, due to different stages of viral replication and host immune responses with significant difference of gene expression, protein modifications, and other molecular processes. This evidence enlightens us to consider different stages of viral infection for developing novel antiviral strategies. (Q3,Q6)

#### 3.3.2 Apoptosis

As a common manner of programmed cell death, apoptosis maintains the homeostasis of the host's internal environment and plays an indispensable regulatory role in physiological processes such as ontogeny, tissue remodeling, and immune regulation (Obeng, 2021). Depending on the regulatory mechanism, this process can be divided into mitochondria-mediated endogenous pathways and death receptor-mediated exogenous pathways, in which the cascade activation of the effector protease caspase-3 constitutes the common terminal execution link of the two apoptotic pathways (Elmore, 2007). At the host antiviral defense aspect, immune cells remove abnormal viral particles by initiating apoptotic programs to limit the spread of pathogens, but viruses have also developed multiple antagonistic strategies in the process of evolution, breaking through the host defense barrier by interfering with molecular mechanisms such as caspase activation or inhibiting apoptotic signal transduction, thereby establishing favorable conditions for their replication cycle in the early stage of infection (Zhou et al., 2024).

PEDV infection triggers PINK1/Parkin-mediated mitophagy by ubiquitinating MFN2 via its N protein, which suppresses early apoptosis and inhibits the JAK1-STAT1 signaling pathway to promote viral replication and immune evasion (Li et al., 2025). Ding L. et al. (2014) validated that TGEV N protein may play an important role in TGEV infection-induced p53 activation process, as well as cell cycle arrest and apoptosis in S and G2/M phases. Myeloid cell leukemia-1 (MCL-1) is an anti-apoptotic BCL-2 family protein that controls the initiation of the apoptotic program by regulating the mitochondrial pathway in endogenous apoptosis (Nagata and Tanaka, 2017; Cory and Adams, 2002). SARS-CoV-2 N protein promotes viral replication by inhibiting MCL-1-dependent apoptosis (Pan et al., 2023). IBV N protein can induce the accumulation of reactive oxygen species (ROS) in HD11 cells and induce apoptosis and affect viral replication (Han et al., 2024).

#### 3.3.3 Pyroptosis

Pyroptosis is an inflammatory cell death that serves as a defense mechanism for the host against pathogen invasion (Churchill et al., 2022). Moreover, pyroptosis serves as an important approach within the innate immune responses, inducing PAMPs and damage-associated molecular patterns (DAMPs) in response to pathogen invasion (Yu et al., 2021). Macrophages receive PAMPs and DAMPs signaling to induce the formation of the NOD-like receptor pyrin-domain-containing 3 (NLRP3) inflammasome. NLRP3 is highly expressed in the gut and is strongly associated with immune and inflammatory responses (Mei et al., 2019). Upon activation of NLRP3, the adaptor protein ASC forms multi-subunit aggregates, which in turn activates caspase-1, which can promote the cleavage of gasdermin D (GSDMD). GSDMD is the executioner of pyroptosis and is mainly expressed in the skin, immune cells, and gastrointestinal tissues. Lysed GSDMD forms membrane pores, leading to cytokine release and pyroptosis (Burdette et al., 2021). It is clear that triggering pyroptosis and inflammasome activation is detrimental to viral replication and survival (Kanneganti et al., 2006). As a result, viruses have also developed strategies to antagonize generalized inflammasome activation and pyroptosis. Pan et al. reported that SARS-CoV-2 N induces a cytokine storm by promoting the assembly and activation of NLRP3 inflammasomes, influencing host cell pyroptosis, thereby providing a favorable environment for virus transmission (Pan et al., 2021). SARS-CoV-2 N protein can directly bind to GSDMD, thereby suppressing its cleavage by caspase-1 and inhibiting pyroptosis. As a result of impaired pyroptosis, infected cells do not undergo rapid rupture and death, which facilitates viral persistence and ongoing replication within the host cells. This mechanism effectively prolongs the viral replication window (Ma et al., 2021). (Q6)

### 3.4 N protein in regulating cell cycle

Coronavirus N protein also plays a key role in regulating the host cell cycle through multiple mechanisms to create favorable conditions for viral replication. As the host p53 is a key protein in cell cycle regulation and DNA damage response, researchers found that the N protein binds to p53, inhibiting its function and thereby interfering with the cell cycle progression (Xu et al., 2016). For example, PEDV N protein utilizes its nuclear localization signal (NLS) for the interaction with p53 in the nucleus, activating the p53-DREAM signaling pathway to induce the S-phase arrest and promote the viral replication (Su et al., 2021). Since p53 is reported to be an antiviral host factor, PEDV N protein could recruit the E3 ubiquitin ligase COP1 to inhibit COP1 self-ubiquitination and degradation, thus facilitating COP-1-mediated ubiquitination-dependent proteasomal degradation of p53 (Dong et al., 2024). Except for p53, Zhu et al. (2022) also screened the protein expression profiles of the PEDV N protein-induced S-phase arrested cells and found most of the differentially expressed proteins are associated with DNA replication, RNA transcription and protein synthesis, indicating that coronavirus may facilitate its own replication by utilizing ribosome proteins associated with protein synthesis involved in the cell cycle. For the SARS-CoV-2 infection, its N protein interacts with Smad3 to activate TGF-β-Smad3 pathway and the downstream Smad3-dependent G1 cell cycle arrest for acute kidney injury (Wang et al., 2022). Therefore, these clues illustrate that coronavirus N protein creates favorable conditions for the viral replication and infection in the host cells by disturbing the cell cycle. These mechanisms provide important references for understanding the role of the N protein in viral infection and pathogenicity.

### 3.5 N protein regulates post-translational modifications for immune evasion

Post-translational modifications (PTMs) refer to the covalent modification of a protein synthesized by a specific enzyme to add the corresponding functional group in the form of a covalent bond to one or more amino acid residues. These modifications play an important role in regulating protein activity, stability, subcellular localization, and mediating protein interactions. PTMs include phosphorylation, succinylation, methylation, acetylation, etc. During the interaction between N protein and host, PTMs of N protein promotes viral replication, assembly, release, and inhibition of IFN response, thereby promoting viral proliferation and immune escape (Wu et al., 2023; Cheng et al., 2022).

During PEDV infection, the PEDV N protein enhances STAT1 acetylation by downregulating deacetylase (HDAC1), subsequently blocking STAT1 phosphorylation and nuclear translocation, evading the host's innate immune response by inhibiting antiviral IFN signaling (Xu et al., 2023). Phosphorylation of coronaviruses N proteins is associated with glycogen synthase kinase 3 (GSK3). Studies have shown that GSK3α/β plays a major role in the SR motif of phosphorylated N protein. Phosphorylation of the SR motif of N protein promotes the proliferation of PEDV (Jia et al., 2025). Wu et al. showed that GSK-3 inhibition reduces SARS-CoV N protein Ser177 phosphorylation and viral replication, indicating this modification's importance for viral proliferation (Wu et al., 2009). The N protein of SARS-CoV-2 binds to the host GSK-3β via the GSK-3 interaction domain (GID), which plays a key role in N protein phosphorylation and viral replication (Yun et al., 2022). In terms of phosphorylation modification, it was found that the phosphorylation modification of SARS-CoV-2 N protein can enhance its affinity for RNA binding, promote viral replication and viral particle assembly, and interfere with the localization of stress granules in host cells (Carlson et al., 2020; Lu et al., 2021). Simultaneously, Carlson et al. confirmed that N protein phosphorylation sites (S176, S180, S184, S197, S206, T205) are the core targets for regulating N protein phase separation and functional switch. The low phosphorylation state of N protein promotes RNA packaging, while the high phosphorylation state shifts to virion assembly (Carlson et al., 2020). N protein can also block the phosphorylation of IRF3 and TBK1 in the JAK-STAT signaling pathway, thereby inhibiting the host immune response (Zhao et al., 2021). It is also reported that binding of phosphorylated SARS-CoV N^S177^ and N^S180^ to the host 14-3-3 protein in the cytoplasm was regulated nucleocytoplasmic N shuttling (Surjit et al., 2005). Tugaeva et al. reported that the binding of the N protein dimer of SARS-CoV-2 to the “bridge protein” 14-3-3 dimer requires serine phosphorylation at position 197 of the N protein, and that the interaction between 14-3-3 and the N protein can affect viral replication (Tugaeva et al., 2021). Tomer M et al. found that phosphorylation modification of SRAS-CoV-2 N protein requires the SR-specific protein kinase SRPK1/2 to act, thereby triggering further phosphorylation of GSK3α/β and CK1 to achieve broad phosphorylation of the SR-rich domain of N protein. Treated with the kinase inhibitor Alectinib, it is able to inhibit N phosphorylation of SRPK1/2 and restrict SARS-CoV-2 replication (Yaron et al., 2020). In 2022, Liu et al. reported that SARS-CoV-2 protein succinylation mainly occurs on M protein and N protein. K65 and K102 are situated within the RNA-binding domain of the N protein, while acetylation sites such as K266 are located in or near the dimerization region, potentially influencing N protein oligomerization. Succinylation of the N protein enhances viral replication by modulating host cell metabolism, including the accumulation of tricarboxylic acid (TCA) cycle intermediates (Liu et al., 2022). Cai et al. (2021) found that SARS-CoV-2 N proteins can also be methylated, with the methylation of the N proteins R95 and R177, the methylation of R95 inhibiting the formation of host cell stress granules and thus affecting the antiviral response, while the methylation of R177 promotes the binding of N protein to RNA and promotes viral replication. Acetylation of SARS-CoV-2 N protein at K61 impairs RNA-binding capacity and compromises viral assembly. CREB-binding protein (CBP)-mediated acetylation at K375 further demonstrates the regulatory role of this modification in the viral life cycle, highlighting potential antiviral strategies targeting N protein acetylation (Hatakeyama et al., 2021). Zhu et al. (2024) revealed that the N protein of SARS-CoV-2 is ISGylated by the HERC5 ISGylation mechanism, a modification that hinders the assembly of N functions into oligomers, ultimately inhibiting viral RNA synthesis and the ISGylation levels of N are controlled by direct deISGylation by NSP3 papain-like protease (PLpro) enzymatic activity. Chen et al. identified phosphorylation sites adjacent to the RNA-binding domain of IBV N protein using mass spectrometry. Surface plasmon resonance (SPR) kinetic analysis further demonstrated that phosphorylated N protein exhibits enhanced specificity for viral RNA recognition (Chen et al., 2005). The above studies show that PTMs play an important role in the interaction between viruses and hosts, and these PTMs of N protein may become potential targets for future antiviral drug development (Table 1).

**Table 1:** The functional mechanisms of different PTMs on coronavirus N proteins.

**Viruses**	**Related PTMs**	**Modification sites**	**Functional mechanisms**	**References**
PEDV	Phosphorylation	STAT1 phosphorylation (unknown)	N protein regulates STAT1 acetylation-phosphorylation switch, resulting in inhibition of the antiviral IFN signaling pathway.	Xu et al., 2023
PEDV	Phosphorylation	Unknown	N protein SR motif phosphorylation by GSK3α/β promotes the PEDV proliferation.	Jia et al., 2025
SARS-CoV	Phosphorylation	S177, S180	The N protein downregulates the expression of 14-3-3 protein, resulting in the accumulation of phosphorylated N protein in the cell nucleus.	Surjit et al., 2005
SARS-CoV	Phosphorylation	S177	GSK-3-regulated N^S177^ phosphorylation promotes RNA binding, oligomerization, and immune escape function of N proteins.	Wu et al., 2009
SARS-CoV-2	Phosphorylation	Unknown	GSK-3-mediated N phosphorylation is required for its structural loading in a virus-like particle (VLP).	Yun et al., 2022
SARS-CoV-2	Phosphorylation	Unknown	N protein phosphorylation of the enhanced affinity for interfering host stress granules, RNA binding, promoting viral replication and viral particle assembly.	Lu et al., 2021
SARS-CoV-2	Phosphorylation	S197	The tight binding of 14-3-3 to phosphorylated N protein may regulate the function of N protein by masking the SR region, and may also affect the host pathway by hijacking 14-3-3.	Tugaeva et al., 2021
SARS-CoV-2	Phosphorylation	Unknown	Alectinib disrupts N protein phosphorylation by SRPK1/2, resulting in viral RNP assembly and replication suppression.	Yaron et al., 2020
SARS-CoV-2	Phosphorylation	S176, S180, S184, S197, S206, and T205	The low phosphorylation state of N protein promotes RNA packaging, while the high phosphorylation state shifts to virion assembly.	Carlson et al., 2020
SARS-CoV-2	Succinylation	K65, K102	Affect the dimerization of N protein and metabolic pathways.	Liu et al., 2022
SARS-CoV-2	Methylation	R95, R177	The N protein methylated by PRMT1 inhibits viral replication and enhances the antiviral ability of host cells.	Cai et al., 2021
SARS-CoV-2	Acetylation	K61, K375	N protein acetylation by CREB-binding protein (CBP) reduces virulence.	Hatakeyama et al., 2021
SARS-CoV-2	ISGylation	K266, K355, K387, and K388	N protein ISGylation by HERC5 hinders the assembly of N into oligomers, ultimately inhibits viral RNA synthesis.	Zhu et al., 2024
IBV	Phosphorylation	S190	N phosphorylation reduces the ability to bind to RNA by altering the charge distribution or conformation of N proteins.	Chen et al., 2005

## 4 Conclusion and perspectives

In summary, the coronavirus N protein is a structurally complex and functionally versatile molecule that engages in extensive interactions with host proteins throughout the viral life cycle. By modulating innate immunity, programmed cell death, and cell cycle regulation, which often through diverse post-translational modifications, N protein exerts profound effects on host-virus interactions. The extensive evidence reviewed in this review solidifies the coronavirus N protein as a master regulator of viral replication and a central antagonist of the host innate immune system. Beyond its canonical role in genome packaging, the N protein strategically targets multiple nodes of the antiviral response, from pathogen sensing to effector function. These multifaceted roles strongly suggest that coronavirus N proteins represent a promising and underexplored target for the development of antiviral drugs and vaccines. Its high sequence conservation across coronaviruses and essential role in the viral life cycle make it an attractive candidate for countering future zoonotic threats and variant strains.

Previous antiviral strategies have largely focused on structural proteins such as the S protein or on enzymatic proteins like RNA-dependent RNA polymerase. While these approaches have yielded valuable therapeutic candidates, their efficacy is often limited by rapid viral mutation and immune evasion. In contrast, the N protein is highly conserved across coronaviruses, which makes it an attractive target for the development of broad-spectrum anti-coronavirus therapeutics. The conserved structural basis of NTD-RNA interaction and different viral proteins interaction (e.g., N-M interaction), might be a potential target for design of small molecule drugs. Also, the landscape of N protein PTMs in coronaviruses has not been comprehensively mapped, demanding systematic phosphoproteomic and ubiquitinomic profiling to decode their regulatory functions. By systematically demonstrating this profile, the antiviral coronavirus therapeutics could also be supported by this specific characteristic of N protein showing a number of amino acids with PTMs. (Q18)

Recent advances in drug discovery, including structure-based design, high-throughput screening, and computational modeling, provide new opportunities to exploit N protein functions for therapeutic intervention.

Moving forward, our research will prioritize determining the authentic crystal structure of the N protein to better reveal its conformational dynamics. On this basis, we aim to elucidate the comprehensive protein-protein interaction networks mediated by N protein and to identify novel post-translational modification sites on both viral and host factors. These efforts will not only deepen the mechanistic understanding of N protein-driven immune evasion but also facilitate the rational design of small-molecule inhibitors, peptide mimetics, or vaccine candidates that specifically target conserved structural and functional domains of N protein. Ultimately, by integrating insights from both previous practices and emerging approaches, this line of research will contribute to the development of broad-spectrum anti-coronavirus drugs, thereby enhancing preparedness for future coronavirus outbreaks.
